# Exploring the nonlinear relationship between robotics manufacturing and urban carbon emissions

**DOI:** 10.1038/s41598-026-46922-y

**Published:** 2026-04-01

**Authors:** Jie Lin, Yizhi Xie, Jianfu Shen

**Affiliations:** 1 Business School, Shantou University, 5 Cuifeng Road, Shantou, 515821 Guangdong China; 2 Department of Social Science and Policy Studies, Education University of Hong Kong, Ting Kok, Hong Kong China; 3 Department of Building and Real Estate, Hong Kong Polytechnic University, Hung Hom, Hong Kong China

**Keywords:** Robotics manufacturing industry, Carbon emissions, Inverted U-shaped relationship, Energy efficiency, Environmental sciences, Environmental social sciences

## Abstract

Robotics is widely regarded as a key driver of digital transformation and green industrial upgrading, yet its environmental implications remain ambiguous. Existing studies primarily focus on the emission effects of robot applications, while largely neglecting robotics manufacturing as an energy- and resource-intensive industrial activity. Using panel data from 277 Chinese prefecture-level cities from 2008 to 2019, this study examines the relationship between robotics manufacturing development and urban carbon emissions. We find a robust inverted U-shaped relationship: carbon emissions initially increase with the expansion of robotics manufacturing but decline once the industry reaches a moderate scale. Mechanism analysis reveals a stage-dependent sequential pathway, whereby mature robotics manufacturing promotes robot adoption, improves urban energy efficiency, and ultimately reduces carbon emissions, while this channel is inactive at early stages. Heterogeneity analysis shows that carbon-mitigation effects are more pronounced in the central region than in the eastern region, suggesting a latecomer advantage in green industrialization. Subsector analysis further indicates that system integration delivers earlier and stronger carbon-reduction effects than ontology manufacturing. These findings highlight the importance of considering industrial life-cycle stages and value-chain positions when designing policies to align high-tech industrial development with carbon-reduction goals.

**Supplementary Information:**

The online version contains supplementary material available at 10.1038/s41598-026-46922-y.

## Introduction

 Since the Industrial Revolution, economic growth and industrial expansion have been closely associated with energy-intensive production systems, resulting in a sustained rise in greenhouse gas emissions^1,2^. Excessive carbon dioxide (CO₂) emissions have become one of the most pressing global challenges, exacerbating climate change and threatening human health and ecological sustainability^3^. For developing economies such as China, where sustaining industrial growth remains a fundamental development objective, the core challenge lies in reconciling economic expansion with carbon-reduction targets. Despite a recent slowdown in the growth of energy-related emissions, China still accounted for 32.8% of global CO_2_ emissions in 2022 and has remained the world’s largest emitter since 2005.^1^ This pattern is largely driven by its energy-intensive secondary sector, which consumes over 70% of total energy and generates around 80% of energy-related emissions^1^. Against this background, identifying industrial transformation pathways that enable carbon mitigation without undermining industrial competitiveness has become a defining issue of sustainable development.

To address this challenge, the Chinese government has implemented a series of industrial restructuring and environmental governance policies aimed at improving energy efficiency and promoting green transformation. The *14th Five-Year Plan for Green Development of Industry* emphasizes a dual strategy: accelerating the low-carbon upgrading of traditional industries while fostering strategic emerging industries, particularly high-end equipment manufacturing.^2^ Within this policy framework, the robotics industry has been positioned as a key pillar supporting both digitalization and green industrial transformation, highlighting its potential to reshape production systems and influence urban carbon emissions.

As a representative high-tech industry, robotics integrates advanced technologies such as artificial intelligence, machine learning, and sensor systems, and plays a pivotal role in modern production systems^4^. On the one hand, robotics manufacturing itself is a rapidly expanding industrial sector characterized by capital intensity, complex production processes, and substantial energy demand^5^. On the other hand, the diffusion and application of robotic technologies across manufacturing and service sectors can enhance energy efficiency, optimize production processes, and reduce waste and emissions^6–8^. This dual nature implies that the environmental consequences of robotics development are theoretically ambiguous rather than uniformly beneficial.

From a theoretical perspective, the impact of robotics manufacturing on carbon emissions operates through two interrelated channels. First, the production-side channel reflects the carbon emissions generated during robot manufacturing, including energy consumption in production processes, upstream material inputs, and industrial agglomeration effects. During the early stages of industry expansion, rapid scale enlargement and clustering may intensify energy use and raise carbon emissions. Second, the application-side channel captures the indirect emission-reduction effects arising from the diffusion of robotic technologies, which can facilitate industrial upgrading, improve energy efficiency, and promote cleaner production practices across downstream industries^9^. Importantly, the relative strength of these two channels evolves dynamically as the industry matures.

Drawing on the theoretical logic of the Environmental Kuznets Curve (EKC), industrial life-cycle theory, and innovation diffusion theory, this study conceptualizes robotics manufacturing as a dynamic process characterized by shifting dominance among scale effects and technology effects^10,11^. In the early stage, scale expansion and industrial agglomeration tend to dominate, leading to rising emissions. As the industry advances, industrial upgrading and technological innovation gradually strengthen, enhancing energy efficiency and offsetting initial pollution effects^12,13^. This dynamic evolution predicts a nonlinear—specifically inverted U-shaped—relationship between robotics manufacturing development and urban carbon emissions.

Despite the growing literature on robots and environmental outcomes, existing studies predominantly focus on the application effects of robots, treating robotic adoption as an exogenous technological shock that enhances productivity and reduces emissions through efficiency gains^9^. Far less attention has been paid to robotics manufacturing as an endogenous and rapidly expanding industrial sector with its own emission characteristics. Moreover, prior research rarely distinguishes or systematically links the environmental impacts across different segments of the robot industrial chain, thereby overlooking the interaction between production-side pollution and application-side efficiency improvements. This omission limits our understanding of the true environmental consequences of robotics development at the urban level.

Against this backdrop, this study empirically examines the relationship between robotics manufacturing development and urban carbon emissions using panel data from 277 Chinese cities over the period 2008–2019. The results reveal a robust inverted U-shaped relationship: carbon emissions initially increase with the expansion of robotics manufacturing but decline as technological maturity, industrial upgrading, and efficiency improvements take effect. Mechanism analysis shows that robotics manufacturing accelerates robot adoption, which in turn enhances urban energy efficiency and constitutes the primary transmission channel. Heterogeneity analysis further indicates that emission-reduction effects are more pronounced in central and western regions than in eastern China, and that the system-integration segment of the robotics industry exhibits stronger carbon-reduction effects than the more energy- and material-intensive ontology manufacturing segment.

This study makes three main contributions. First, it extends the EKC framework by shifting the analytical focus from aggregate economic growth to a specific strategic emerging industry—robotics manufacturing—and provides novel empirical evidence on its relationship with urban carbon emissions. Second, it refines the understanding of nonlinear environmental effects of high-tech industries by showing that carbon-mitigation effects emerge gradually as robotics manufacturing evolves from scale expansion toward technological diffusion, with pronounced regional and subsectoral heterogeneity. Third, drawing on new economic geography, this study integrates carbon emissions into the analysis of high-tech industrial agglomeration, thereby enriching the environmental dimension of agglomeration economics.

## Literature review and hypothesis development

### Impacts of high-tech industries on carbon emissions

There is an ongoing debate regarding whether the development of high-tech industries contributes to carbon emission reduction and sustainable development. A large body of empirical research suggests that high-tech industries can promote technological innovation, enhance energy efficiency, and thereby reduce carbon emissions (Xu and Lin^14,15^. Such effects have been documented across a range of sectors, including information and communication technologies (ICT)^16,17^, artificial intelligence (AI)^7,18^, and new energy vehicles^19^. For instance, Haini^20^, finds that ICT development is associated with declining carbon emissions in ASEAN economies, while Liu et al.^7^, show that AI-related technological progress significantly suppresses carbon intensity, albeit with notable heterogeneity across sectors and development stages. Related studies further emphasize that spatial agglomeration and knowledge spillovers can amplify the emission-reduction effects of high-tech industries at both local and regional levels^21^.

At the same time, a growing strand of literature highlights potential countervailing effects: high-tech industry expansion may increase overall energy consumption, generating rebound effects that offset efficiency gains^22–24^. Lange et al., (2020) show that although ICT development improves energy efficiency and promotes industrial upgrading, total energy consumption continues to rise with sectoral expansion. Similarly, Strubell et al.^25^, point to the substantial carbon footprint associated with training large-scale AI models, while Wen et al.^26^, identify persistent energy-efficiency challenges in smart manufacturing.

Beyond these mixed findings, some studies propose a nonlinear relationship between high-tech industry development and carbon emissions. Drawing on the Environmental Kuznets Curve (EKC) logic, existing evidence suggests that emissions may increase during early development stages due to infrastructure construction and production expansion, but decline later as technological maturity and efficiency improvements take effect (Xu and Lin^27,28^,. This literature indicates that the environmental impacts of high-tech industries are dynamic rather than monotonic. However, whether these general patterns apply to specific strategic emerging industries—such as robotics manufacturing—remains unclear.

### Robotics applications and carbon emissions

Compared with the extensive literature on high-tech industries more broadly, studies explicitly examining robotics and carbon emissions remain relatively limited. Existing robotics-related research has predominantly focused on the application stage of robotic technologies, treating robots primarily as productivity-enhancing inputs adopted by firms in manufacturing and related sectors^29^. A substantial body of evidence shows that robot applications can accelerate digital and intelligent transformation, stimulate process innovation, and improve productivity, thereby enhancing energy efficiency and reducing carbon intensity^9,30–32^.

Nevertheless, the literature on robot applications also reports mixed environmental outcomes. Some studies suggest that large-scale robot deployment may increase energy demand during the transition to automation, driven by higher electricity consumption and expanded production capacity^33^. As a result, the net carbon effects of robot applications remain context-dependent and potentially nonlinear.

Notably, this strand of research typically conceptualizes robots as exogenous technological shocks, with their environmental impacts arising primarily through adoption and use. As a result, existing research largely overlooks the potential environmental impacts originating from the manufacturing stage of robotics itself. Consequently, the environmental implications associated with robotics manufacturing—distinct from those of robot applications—remain insufficiently explored, leading to an incomplete assessment of the environmental impacts of the robotics industry.

### Robotics manufacturing: a neglected dimension of carbon emissions research

Robotics manufacturing represents a distinct and rapidly expanding high-tech industrial activity whose environmental implications primarily stem from its characteristics as an energy- and resource-intensive production process. As part of the equipment and instrument manufacturing sector, robotics manufacturing involves activities such as component fabrication, system assembly, and supply-chain integration, all of which require substantial inputs of electricity and materials^5^. From this perspective, the environmental consequences of robotics manufacturing differ fundamentally from those associated with robot applications. At the same time, robotics manufacturing also shapes application-side outcomes by enabling the diffusion and use of robotic technologies across industries, thereby introducing an additional channel through which carbon effects may arise. As a result, the carbon emission impacts of robotics manufacturing are inherently multifaceted rather than unidirectional.

From a micro-theoretical perspective, the carbon emission impact of robotics manufacturing can be understood as the net outcome of two opposing forces: a scale effect and a technology effect. The scale effect originates primarily from the expansion of robotics manufacturing activities themselves. In the early stage of industry development, the entry of manufacturing firms, the construction of production facilities, and the rapid enlargement of manufacturing capacity intensify the use of energy and material inputs. These early-stage manufacturing-side dynamics lead to rising carbon emissions, as efficiency improvements are initially insufficient to offset the energy demand associated with industrial expansion.

In contrast, the technology effect operates through two complementary channels that become increasingly important as the industry matures. First, within the robotics manufacturing sector, technological upgrading is reinforced by spatial agglomeration and industrial synergy. In China, robotics manufacturing enterprises—including component producers, ontology manufacturers, and system integrators—have increasingly clustered in regions such as the Yangtze River Delta, the Pearl River Delta, and the Beijing–Tianjin–Hebei area, as well as in emerging manufacturing cities such as Wuhan, Hefei, Changsha, and Chongqing^34^. Such agglomeration facilitates knowledge spillovers, collaborative innovation, and the diffusion of energy-efficient production technologies within the manufacturing system^35,36^, thereby reducing energy consumption per unit of output and strengthening the technology effect on the manufacturing side.

Second, as robotics manufacturing capabilities continue to advance, technological progress within the sector supports the application-side diffusion of robotic technologies across a wide range of industries. Localized concentrations of robotics manufacturers—particularly system integration firms—lower customization costs, enhance technical support, and reduce adoption barriers for user industries, including small and medium-sized enterprises^30,37^. Importantly, these diffusion-related efficiency gains are not immediate but tend to materialize at more advanced stages of adoption, once learning effects accumulate and application costs decline. Through this channel, robotics manufacturing indirectly contributes to improvements in production precision, continuity, and energy efficiency in user industries^4^, further reinforcing the overall technology effect.

Taken together, the dominance of the scale effect in the early stage of robotics manufacturing development and the growing strength of technology effects—both within manufacturing and through application-side diffusion—imply a dynamic and nonlinear relationship between robotics manufacturing and carbon emissions. In the initial phase, manufacturing expansion drives emissions upward; as the industry matures, technological upgrading and diffusion-related efficiency gains increasingly offset and eventually surpass the scale effect. This theoretical framework provides a clear micro-foundation for an inverted U-shaped relationship between robotics manufacturing development and urban carbon emissions. Accordingly, this study proposes the following hypothesis:

#### H1

The influence of the robotics manufacturing industry on urban carbon emissions exhibits an inverted U-shaped relationship.

### Mechanisms of robotics manufacturing industry on carbon emissions

We further investigate the transmission pathway through which the development of the robotics manufacturing industry influences carbon emissions. The existing literature consistently suggests that improvement of energy efficiency serve as an important mechanism by which high-tech industries achieve emission reductions^9,31,38^. This view is grounded in the fact that the growth of high-tech industries is typically accompanied by technological innovation, reduced dependence on fossil fuels, and the adoption of more efficient energy-conversion technologies^39,40^. Empirical studies support this pathway: Schulte et al.^17^, show that high-tech industries reduce both total and non-electric energy demand cross OECD countries, while Morrison and Golden^41^demonstrate that the bioenergy high-tech sector in the United States can effectively reduce coal consumption and thereby lower CO₂ emissions. More recently, Yu et al.^9^, provide evidence that high-tech products, particularly industrial robots, reduce urban carbon emissions by enhancing energy efficiency.

Beyond this general mechanism, robotics manufacturing exhibits a distinctive capacity to influence regional energy efficiency because robotics is widely regarded as a general purpose technology with broad applicability and strong complementarities with existing production systems^42^. The realization of such system-wide efficiency gains depends not only on technological progress within the manufacturing sector itself, but also on how effectively robotic technologies are adopted and integrated across the local economy. In this respect, the localization of robotics manufacturing—particularly the presence of system integrators and specialized suppliers—can lower adoption barriers by enabling customization, technical support, and iterative learning between producers and users, thereby facilitating the diffusion of robotic technologies. Accordingly, the first step of the transmission process concerns whether the development of robotics manufacturing promotes the adoption and diffusion of robotic technologies within the regional economy.

The second step concerns how the diffusion of robotic technologies affects regional energy efficiency. By improving production precision, process coordination, and operational continuity, robotic adoption can reduce energy consumption per unit of output. More importantly, when adoption becomes sufficiently widespread, efficiency gains may extend beyond individual firms through demonstration effects, inter-firm learning, and cross-industry spillovers, generating measurable improvements in regional overall energy efficiency.

Finally, improvements in regional energy efficiency are expected to translate into lower carbon emissions by reducing energy intensity and optimizing energy use across industries. While robotics manufacturing may also influence carbon emissions through other channels, the above reasoning highlights an ordered influence process in which energy efficiency driven by adoption plays a central mediating role. This leads to the following hypothesis:

#### H2

Robotics manufacturing reduces urban carbon emissions by promoting the diffusion of robotic technologies and thereby enhancing regional energy efficiency.

## Methodology

### Regression model

Considering the dual effects of unobservable heterogeneity across cities and annual variation, we adopt a two-way fixed-effects model for our empirical analysis to control for potential confounding factors. The baseline model is specified as follows:

1 $$\:{lnCO2}_{it}={\beta\:}_{0}+{\beta\:}_{1}{lnSRobot}_{it}+{\beta\:}_{2}({{lnSRobot}_{it})}^{2}+\sum\:{\beta\:}_{3}{X}_{it}+{\mu\:}_{i}+{v}_{t}+{\epsilon\:}_{it}$$

where $\:i$ and $\:t$ represent the city and year, respectively; $\:{lnCO2}_{it}$ denotes the explained variable, which is the logarithmic form of CO_2_ emissions of city $\:i$ in year $\:t$; $\:{lnSRobot}_{it}$ is a core explanatory variable indicating the development level of the robotics manufacturing industry of city $\:i$ in year $\:t$; *(*$\:{lnSRobot}_{it})$^2^ is the quadratic term of $\:{lnSRobot}_{it}$; $\:{X}_{it}$ denotes a range of control variables; $\:{\mu\:}_{i}$ and $\:{v}_{t}$ are the city and year fixed effects, respectively; and $\:{\epsilon\:}_{it}$ is the residual.

The coefficient of the quadratic term *(*$\:{lnSRobot}_{it})$^2^, denoted as $\:{\beta\:}_{2}$, captures the non-linear relationship between the development level of the robotics manufacturing industry and carbon emissions. The key term of interest is the extreme point of the quadratic formula, which represents the level of robotics manufacturing development at which the relationship between robotics manufacturing and carbon emissions shifts from positive to negative (or vice versa). This extreme point can be calculated as:

2 $$\:Extreme\:point=\frac{-{\beta\:}_{1}}{2{\beta\:}_{2}}$$

This extreme point is crucial to our analysis, as it marks the threshold at which the environmental impact of robotics manufacturing changes direction.

To test **H2**, this study examines the mediating roles of robot adoption and energy efficiency in the relationship between robotics manufacturing and carbon emissions. The mechanism analysis follows a stepwise regression framework based on the approach proposed by Baron and Kenny^43^, supplemented by a Bootstrap test to assess the significance of the mediation effect.

First, we estimate the effect of robotics manufacturing on robot adoption:

3 $$\:{lnadoption}_{it}={\gamma\:}_{0}+{\gamma\:}_{1}{lnSRobot}_{it}+\sum\:{\gamma\:}_{3}{X}_{it}+{\mu\:}_{i}+{v}_{t}+{\epsilon\:}_{it}$$

Second, we examine whether robotics manufacturing and robot adoption jointly affect regional energy efficiency:

4 $$\:{EE}_{it}={\delta\:}_{0}+{\delta\:}_{1}{lnSRobot}_{it}+{\delta\:}_{2}({{lnSRobot}_{it})}^{2}+{{\delta\:}_{3}lnadoption}_{it}+\sum\:{\delta\:}_{4}{X}_{it}+{\mu\:}_{i}+{v}_{t}+{\epsilon\:}_{it}$$

Finally, we incorporate both robot adoption and energy efficiency into the carbon-emission equation to test the full mediation pathway:

5 $$\:{lnCO2}_{it}={\theta\:}_{0}+{\theta\:}_{1}{lnSRobot}_{it}+{\theta\:}_{2}({{lnSRobot}_{it})}^{2}+{\theta\:}_{3}{EE}_{it}+{\theta\:}_{4}{lnadoption}_{it}+\sum\:{\theta\:}_{4}{X}_{it}+{\mu\:}_{i}+{v}_{t}+{\epsilon\:}_{it}$$

where $\:{lnadoption}_{it}$ denotes the intensity of robot adoption, and $\:{EE}_{it}$ represents energy efficiency in city $\:i$ in year $\:t$. The vector $\:{X}_{it}$ includes the same set of control variables as in the baseline model. City fixed effects ($\:{\mu\:}_{i}$) and year fixed effects ($\:{v}_{t}$) are included to control for unobserved heterogeneity, and $\:{\epsilon\:}_{it}$ is the residual.

### Variables

#### Explained variables

The literature uses various indicators to measure carbon emissions, such as total CO_2_ emissions^9^, per capita CO_2_ emissions^44^, and CO_2_ emissions per unit of energy consumption^45^. As our focus is on the impact of the robotics manufacturing industry on total urban carbon emissions, we adopt the logarithm of total CO_2_ emissions $\:\left(lnCO2\right)$ as a key metric for assessing a city’s carbon emission intensity in the baseline regression. As a robustness test, we employ the logarithm of per capita CO_2_ emissions $\:\left(lnPCO2\right)$ to bolster the reliability and resilience of our findings.

#### Core explanatory variables

Following Bin^46^, the number of enterprises is a key indicator of industry development. In high-tech sectors, firm size does not necessarily correspond to economic contribution or technological potential. Accordingly, we construct the core explanatory variable as the stock of robotics manufacturing enterprises in each city and year, expressed in logarithmic form$\:(lnSRobot$). As a robustness check, we replace $\:lnSRobot\:$with$\:\:lncap$, defined as the total registered capital of robotics manufacturing enterprises, which captures the industry’s scale from a capital-based perspective.

#### Mediating variable

This study employs robot adoption and energy efficiency as mediating variables. Robot adoption ($\:{lnadoption}_{it}$) is measured as the number of industrial robots adopted per 10,000 persons, capturing the intensity of robot use at the city level^9^. Energy efficiency ($\:EE$) reflects the effectiveness of energy utilization and its close association with carbon-emission outcomes, and is measured as the ratio of gross domestic product (GDP) to annual industrial electricity consumption^14,31^.

#### Control variables

Urban carbon emissions are influenced by a range of economic, demographic, industrial, and institutional factors beyond the development of the robotics manufacturing industry. Following the literature on urban carbon emissions and green development, we include a set of city-level control variables to mitigate potential omitted-variable bias.

1. Economic development ($\:lnpgdp$): Economic growth generally entails higher energy consumption and is therefore positively associated with carbon emissions^47^.
2. Resident population ($\:lnpop$): Population size is a key determinant of carbon emissions, as population growth increases energy demand for production and consumption^48^.
3. Degree of external openness ($\:lnpfdi$): The environmental impact of foreign direct investment (FDI) is theoretically ambiguous. FDI may reduce emissions through technology spillovers and cleaner production, or increase emissions by attracting pollution-intensive activities^49^. We measure openness using per capita FDI to control for city size.
4. Urbanisation rate ($\:urban$): Urbanization affects carbon emissions through changes in housing, transportation, and industrial agglomeration, and is often associated with higher energy demand in developing economies^50^.
5. Industrial structure ($\:stru$): A higher share of secondary industry typically reflects greater reliance on energy-intensive production processes and is unfavorable to carbon-emission reduction^51^.
6. Energy structure ($\:es$): Energy structure influences carbon-emission intensity through fuel composition. Greater reliance on coal tends to increase emissions. We proxy energy structure by the share of coal consumption in total energy use.
7. Government support for innovation ($\:inno$): Government investment in science and technology can affect emissions by shaping innovation capacity, industrial upgrading, and the diffusion of clean technologies. This variable is measured as the share of science and technology expenditure in total fiscal expenditure.
8. Transportation development *(*$\:lnroad)$: Transportation infrastructure affects emissions through vehicle use and logistics efficiency^9^. We measure transportation development using per capita road area.

Detailed variable definitions and data sources are reported in Table 1, and summary statistics are presented in Table 2. The mean value of $\:lnCO2$ is 6.213 (standard deviation: 1.174), while the average value of the robotics manufacturing development ($\:lnSRobot$) is 1.399. Descriptive statistics for all control variables are also provided.

**Table 1:** Definitions of the variables.

Variable	Definition	Metrics and specifications
lnCO2	Carbon emissions	Logarithm of total carbon emissions
lnPCO2	Per capita carbon emissions	Logarithm of per capita carbon emissions
lnSRobot	Development of the robotics manufacturing industry	Logarithm of the stock of robotic manufacturers
lncap		Logarithm of total registered capital of robotic manufacturers
lnadoption	Density of robot adoption	Logarithm of the number of robots adopted per 10,000 persons.
EE	Energy efficiency	Ratio of GDP to industrial electricity consumption
lnpgdp	Economic development	Logarithm of per capita GDP
lnpop	Resident population	Logarithm of resident population
lnpfdi	Degree of external openness	Per capita Foreign direct investment
urban	Urbanisation	Proportion of urban population in total population
stru	Industrial structure	Proportion of the secondary industry value added in GDP
es	Energy structure	Proportion of coal consumption in total energy consumption
inno	Government support for innovation	Proportion of government science and technology expenditure in total fiscal expenditure
lnroad	Transportation development	Logarithm of per capita road area

**Table 2:** Summary statistics of the variables.

Variable	*N*	Mean	SD	Min	Max
lnCO2	3,324	6.213	1.174	2.117	9.533
lnPCO2	3,324	0.344	1.084	−3.452	4.359
lnSRobot	3,324	1.399	1.769	0.000	6.293
lnFRobot	3,324	4.502	4.580	−6.908	17.744
EE	3,324	24.100	26.880	0.605	886.336
lnadoption	3,324	0.625	1.415	−4.372	3.255
lnpgdp	3,324	10.521	0.620	8.940	11.888
lnpop	3,324	5.869	0.679	3.851	7.582
fdi	3,324	1.766	1.769	0.013	8.573
urban	3,324	53.905	15.206	24.432	94.849
stru	3,324	46.968	10.547	19.409	73.322
es	3,324	81.148	14.468	30.864	98.966
inno	3,324	0.016	0.016	0.001	0.207
lnroad	3,324	2.702	0.442	0.315	4.096

### Research data

This study uses panel data for 277 Chinese prefecture-level cities from 2008 to 2019. The starting year, 2008, marks a turning point in China’s robotics manufacturing development, when industrial robots were designated as priority sectors by central government authorities, signalling strong policy support and the industry’s initial growth phase. The end year, 2019, is chosen to avoid distortions from the COVID-19 pandemic, which caused substantial socio-economic disruptions thereafter. Tibet, Taiwan, Macao, and Hong Kong are excluded from the sample.

Data for this study are drawn from three main sources. First, firm-level information on robotics enterprises is obtained from China’s National Enterprise Credit Information Publicity System (CNECIPS).^3^After removing duplicate records with identical business names, registered addresses, or business scopes, we identify 28,624 enterprises registered between 1 January 2008 and 31 December 2019 and extract information on registration dates, locations, and business scopes. Robot adoption data are sourced from the International Federation of Robotics (IFR) database and constructed following the method of Yu et al.^9^,. urban carbon-emission data are obtained from the China Environmental Statistics Yearbooks. Third, control variables—including GDP per capita, urban population, industrial structure, and other covariates—are collected from the China Urban Statistical Yearbooks.

## Results and discussions

### Inverted U-shaped impact of robotics manufacturing on carbon emissions

The baseline estimation results examining the impact of robotics manufacturing development on urban carbon emissions are reported in Table 3. Column (1) includes only the core explanatory variable $\:{lnSRobot}_{it}\:$and its quadratic term $\:({{lnSRobot}_{it})}^{2}$, while Column (2) incorporates the full set of control variables. Both specifications control for city and year fixed effects. As shown in Column (2), the coefficient of $\:{lnSRobot}_{it}$ is positive and statistically significant at the 1% level (0.054), whereas the coefficient of its squared term is negative and significant at the 1% level (–0.020), indicating a robust inverted U-shaped relationship between robotics manufacturing development and carbon emissions. The U-test strongly rejects the null hypothesis of monotonicity (t = 3.89), confirming the presence of a statistically meaningful nonlinear effect.

Beyond statistical significance, the estimated coefficients also imply economically meaningful marginal effects. Specifically, the marginal effect of a 1% increase in the scale of the robotics manufacturing industry is given by 0.054 − 0.040*$\:{lnSRobot}_{it}$. At relatively low levels of industry development, this marginal effect is positive, indicating that early-stage expansion of robotics manufacturing increases urban carbon emissions, largely due to the energy-intensive nature of production and industrial clustering. As $\:{lnSRobot}_{it}$ increases, however, the negative quadratic term progressively offsets this effect. At the estimated turning point of $\:{lnSRobot}_{it}$ = 1.342, corresponding to approximately four robotics manufacturing enterprises, the marginal effect becomes zero and carbon emissions reach their maximum. Beyond this threshold, the marginal effect turns negative. Evaluated at the sample mean of$\:\:{lnSRobot}_{it}$(1.399), the implied marginal effect is approximately − 0.002, suggesting that a 1% increase in robotics manufacturing scale is associated, on average, with a 0.02% reduction in carbon emissions. This indicates that technological spillovers, production standardisation, and efficiency gains begin to outweigh the initial energy costs once the industry reaches a moderate scale.

**Fig. 1:**
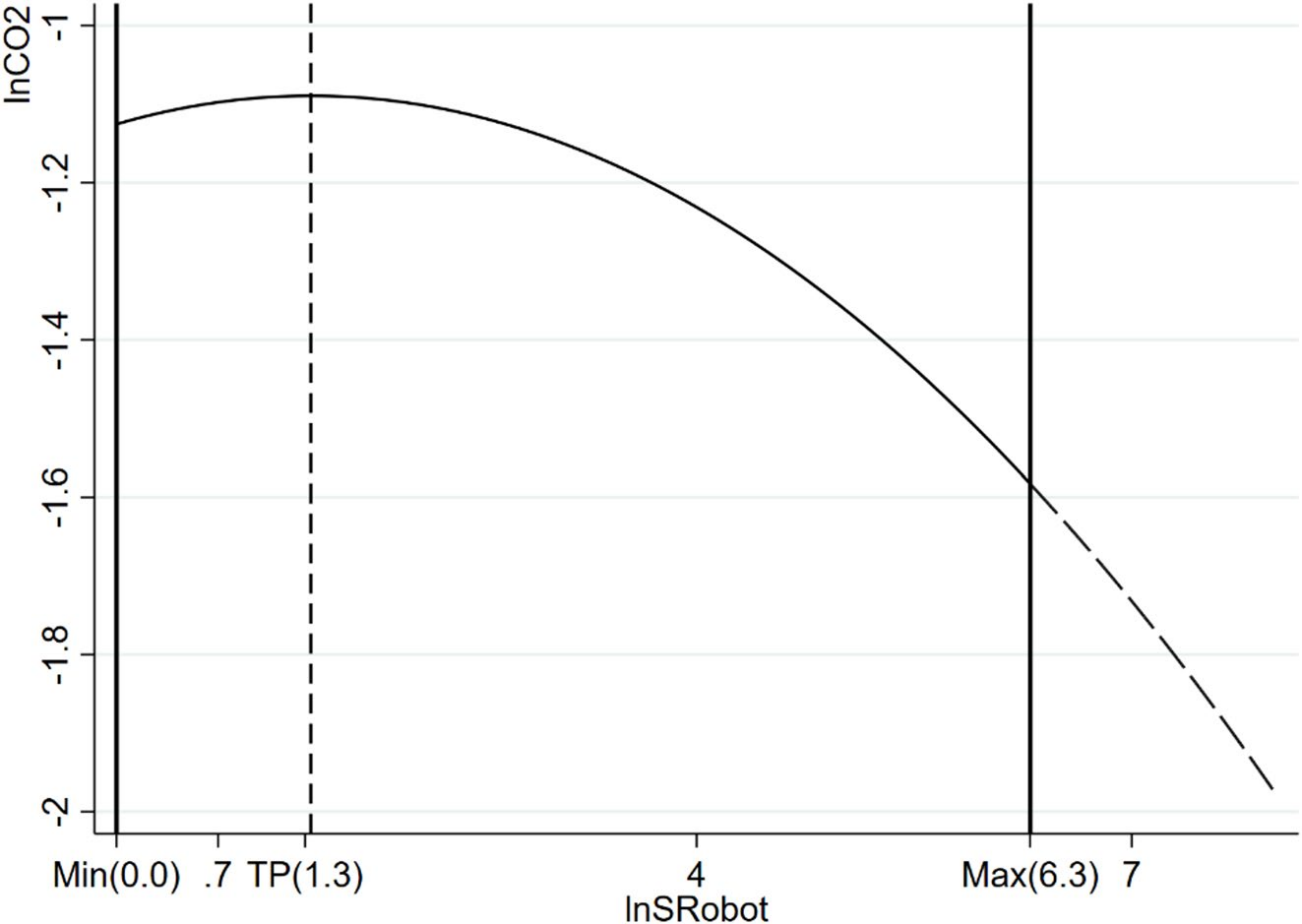
Inverted U-shaped relationships of the robotics manufacturing industry and carbon emissions. *Notes*: Figure 1 illustrates the estimation results incorporating the control variables and city and year fixed effects.

The estimated turning point is relatively low, implying that most cities in the sample already operate on the descending segment of the inverted U-shaped curve. Consequently, for the majority of Chinese cities, further expansion of the robotics manufacturing industry is more likely to contribute to carbon mitigation rather than emission intensification. Figure 1 visualises this nonlinear relationship, showing that robotics manufacturing development raises emissions below the threshold but suppresses emissions once the critical scale is exceeded. This pattern is consistent with the view that early-stage robotics manufacturing is characterised by high energy consumption and limited technological diffusion, whereas large-scale development enhances automation efficiency, optimises production processes, and facilitates cleaner industrial upgrading. The emission-reduction effects of robotics manufacturing are therefore cumulative and path-dependent, requiring sustained industrial expansion rather than short-term interventions.

Regarding the control variables in Column (2), economic development ($\:lnpgdp$), population size ($\:lnpop$), urbanisation ($\:urban$), industrial structure ($\:stru$), energy structure ($\:es$), and the road network density ($\:lnroad)$ all exert significantly positive effects on carbon emissions, in line with existing literature. In contrast, per capita foreign direct investment ($\:lnpfdi$) and government innovation support ($\:inno)$ are statistically insignificant, suggesting no robust direct association with carbon emissions once city and year fixed effects and other covariates are controlled for. Overall, these results indicate that conventional growth-oriented development patterns continue to intensify carbon pressure, underscoring the importance of promoting technology-intensive and efficiency-enhancing industries, such as robotics manufacturing, to achieve long-term emission reductions.

**Table 3:** The results of the baseline estimation.

Dependent variable	(1)	(2)
	lnCO2	
lnSRobot	0.095***	0.054^***^
	(0.014)	(0.014)
(lnSRobot)^2^	−0.027***	−0.020^***^
	(0.004)	(0.004)
lnpgdp		0.136^*^
		(0.081)
lnpop		0.419^***^
		(0.138)
lnpfdi		0.015
		(0.011)
urban		0.024^***^
		(0.004)
stru		0.008^***^
		(0.002)
es		0.017^***^
		(0.002)
inno		−0.367
		(0.709)
lnroad		0.116^***^
		(0.037)
Constant	6.219***	−1.126
	(0.018)	(1.043)
City fixed effect	YES	YES
Year fixed effect	YES	YES
N	3,324	3,324
R^2^	0.910	0.926
Extreme point	1.728	1.342
t-value of the U-test	6.63***	3.89***

Robust standard errors in parentheses, * *p* < 0.1, ** *p* < 0.05, and *** *p* < 0.01.

### Robustness tests and endogeneity mitigation

To assess the robustness of the baseline findings, we conduct a series of robustness checks and endogeneity treatments, including winsorisation of extreme values, alternative specifications of both the explanatory and explained variables, quantile regressions, and an instrumental variable (IV) approach. The corresponding results are reported in Table 4.

First, to mitigate the potential influence of extreme observations, we winsorise the top and bottom 1% of both the core explanatory variable ($\:{lnSRobot}_{it}$) and the explained variable ($\:lnCO2$). As shown in Column (1), the coefficients of$\:\:{lnSRobot}_{it}\:$and its squared term remain positive and negative, respectively, and are statistically significant. This indicates that the inverted U-shaped relationship identified in the baseline regression is not driven by outliers and is robust to distributional trimming.

Second, we examine the sensitivity of the baseline results to alternative variable measurements. Specifically, we replace the number of robotics manufacturing enterprises with the total registered capital of such firms at the city level ($\:lncap$), which captures the quality dimension of industry development by reflecting firms’ scale, capital intensity, and technological commitment. As reported in Column (2) of Table 4, the coefficients of $\:lncap$ and its squared term remain positive and negative, respectively, and are statistically significant, indicating a clear inverted U-shaped relationship. This finding suggests that the nonlinear emission effect is not driven solely by the extensive margin of firm entry but also persists when robotics manufacturing development is measured in terms of capital strength and industry quality. In addition, we redefine the explained variable using per capita carbon emissions ($\:lnPCO2$) to account for differences in city size and population. Column (3) shows that the coefficients of $\:{lnSRobot}_{it}$ and ($\:{lnSRobot}_{it}$)² remain highly significant with unchanged signs, confirming that the inverted U-shaped relationship holds when emissions are measured on an intensity basis rather than in aggregate terms. Together, these results demonstrate that the baseline findings are robust to alternative measurements of both industry development and carbon emissions.

Third, we employ quantile regressions to examine whether the nonlinear relationship varies across cities with different emission levels. As shown in Columns (4)–(8), the linear term of $\:{lnSRobot}_{it}$is positive and the squared term is negative across all quantiles, indicating a consistent nonlinear pattern. However, the U-tests suggest that the inverted U-shaped relationship is statistically significant mainly at the lower and middle quantiles (Q10–Q50), while it becomes insignificant at higher quantiles (Q75 and Q90). This result implies that the emission-reduction effects of robotics manufacturing development are more evident in low- and medium-emission cities, whereas they are weaker or less clearly identified in high-emission cities, pointing to potential heterogeneity across emission levels and motivating further heterogeneity analysis. Finally, to address potential endogeneity concerns, we employ the next year’s stock of robotics manufacturing enterprises ($\:{lnSRobot}_{it+1}$) as an instrumental variable. The relevance of this instrument is supported by the strong path dependence of robotics manufacturing development, as firm establishment and expansion involve long planning horizons and capital commitments, implying a close link between next year’s firm stock and current industrial conditions. Regarding exogeneity, our identifying assumption is that short-term fluctuations in contemporaneous carbon emissions are unlikely to exert a direct causal influence on the number of robotics manufacturing enterprises in the following period, conditional on a rich set of control variables as well as city and year fixed effects. Persistent local development strategies and long-run policy orientations are largely absorbed by city fixed effects, while year fixed effects control for nationwide shocks and common policy trends. Under this framework, $\:{lnSRobot}_{it+1}$ is plausibly exogenous to the contemporaneous error term in the carbon-emission equation. The two-stage least squares (2SLS) estimates reported in Column (9) show that the coefficients of $\:{lnSRobot}_{it}$ and ($\:{lnSRobot}_{it}$)² remain statistically significant and preserve the inverted U-shaped relationship identified in the baseline regressions.

Overall, across multiple robustness checks and alternative estimation strategies, the empirical evidence consistently supports **H1**, confirming a stable inverted U-shaped relationship between the development of the robotics manufacturing industry and urban carbon emissions.

**Table 4:** Results of robustness and endogeneity tests.

	(1)	(2)	(3)	(4)	(5)	(6)	(7)	(8)	(9)
	1% winsorised	Variable substitution		Quantile regressions					IV-2SLS
				Q10	lnCO2	Q50	Q75	Q90	
Explained variable	lnCO2	lnCO2	lnPCO2	0.131*	lnCO2	lnCO2	lnCO2	lnCO2	lnCO2
lnSRobot	0.059^***^		0.055^***^	(0.067)	0.065^***^	0.057	0.050	0.044	0.139^*^
	(0.014)		(0.014)	−0.028***	(0.025)	(0.038)	(0.067)	(0.090)	(0.071)
(lnSRobot)^2^	−0.020^***^		−0.020^***^	(0.008)	−0.024^***^	−0.022^**^	−0.020	−0.019	−0.034^***^
	(0.003)		(0.004)		(0.006)	(0.010)	(0.017)	(0.023)	(0.009)
lncap		0.019^***^							
		(0.006)							
(lncap) ^2^		−0.002^***^							
		(0.001)							
Control variables	YES	YES	YES	YES	YES	YES	YES	YES	YES
City fixed effect	YES	YES	YES	YES	YES	YES	YES	YES	YES
Year fixed effect	YES	YES	YES	YES	YES	YES	YES	YES	YES
N	3,324	3,324	3,324	75.218***	3,324	3,324	3,324	3,324	3,047
R^2^	0.927	0.925	0.913	43.569					
Extreme point	1.471	5.305	1.354	1.388	1.359	1.302	1.238	1.183	
t-value of the U-test	4.33***	2.26***	3.98***	1.974**	2.639***	1.493*	0.736	0.489	
Kleibergen-Paap rk LM statistic									50.190***
Kleibergen-Paap rk Wald F statistic									27.904
Stock-Yogo weak ID test critical values: 10% maximal IV size									7.03

Columns (4)–(8) report standard errors in parentheses. All other columns report robust standard errors in parentheses. * *p* < 0.1, ** *p* < 0.05, and *** *p* < 0.01.

### Sequential mechanism of robot adoption and energy efficiency

The preceding analysis suggests that the emission-reduction effect of robotics manufacturing emerges only after the industry reaches a sufficient scale. To clarify the transmission pathway proposed in Sect. 2.4, we examine a sequential mechanism in which robotics manufacturing development promotes robot adoption, which in turn enhances energy efficiency and ultimately reduces carbon emissions. Following Eq. (3) to (5), we conduct a mediation analysis, with the results reported in Table 5.

Panel A presents the mechanism analysis for the full sample over the entire study period. Column (1) shows that $\:{lnSRobot}_{it}\:$has a significantly positive effect on robot adoption *(*$\:lnadoption$*)*, indicating that the expansion of robotics manufacturing effectively stimulates downstream adoption at the local level. Column (2) further reveals that robot adoption exerts a strong and significantly positive effect on energy efficiency, whereas the direct effect of $\:{lnSRobot}_{it}$ on $\:EE$ is nonlinear—initially negative and turning positive only after surpassing a threshold. This pattern suggests that the effective use of robots in production processes plays a more immediate role in enhancing energy efficiency, while the contribution of robotics manufacturing to efficiency improvements materialises over a longer time horizon.

Column (3) further shows that $\:EE$ has a significantly negative effect on carbon emissions, confirming energy efficiency as an effective emission-mitigation channel. Notably, the estimated turning point of the $\:EE$ response (1.190) occurs earlier than that of carbon emissions (1.342), suggesting a temporal ordering in which improvements in energy efficiency precede and facilitate subsequent emission reductions. The bootstrap test further confirms a statistically significant indirect effect of $\:{lnSRobot}_{it}$ on carbon emissions. Taken together, these results provide consistent evidence that the adoption–energy efficiency pathway constitutes a key channel through which mature robotics manufacturing contributes to carbon reduction.

Given that the baseline results indicate contrasting effects of robotics manufacturing before and after the threshold (1.342), Panels B and C further explore whether this sequential mechanism differs across development stages. Panel B (early stage) shows that the coefficient of $\:{lnSRobot}_{it}$ on lnad is statistically insignificant, indicating that robotics manufacturing has not yet translated into systematic downstream adoption at this stage. As a result, the manufacturing sector does not exert a meaningful influence on energy efficiency through the application-side channel. Although robot adoption itself significantly improves energy efficiency and reduces carbon emissions, such adoption does not originate from local robotics manufacturing. Consequently, the prerequisite link in the mediation chain is absent, and the energy-efficiency mechanism from robotics manufacturing fails to materialize in the early stage. This finding suggests that limited industrial scale constrains the activation of the adoption-driven efficiency channel, even though intelligent production practices remain carbon-reducting once adopted.

In contrast, Panel C (mature stage) provides clear evidence that the sequential mechanism becomes operative once robotics manufacturing surpasses the turning point. Robotics manufacturing significantly promotes local robot adoption, which in turn enhances energy efficiency. Moreover, the negative effect of $\:EE$ on carbon emissions becomes substantially larger in magnitude, indicating that efficiency gains play a more prominent role in emission reduction at this stage. Moreover, the bootstrap-based indirect effect is statistically significant, confirming that the adoption–energy efficiency pathway constitutes a valid transmission channel in the mature phase. This pattern suggests that only after robotic adoption becomes sufficiently widespread do energy-efficiency gains arising from improved production precision, process coordination, and operational continuity diffuse across firms and industries, ultimately reducing carbon emissions.

Overall, the mechanism analysis supports a stage-dependent sequential transmission pathway from robotics manufacturing development to carbon reducstion. The environmental effects of robotics manufacturing are neither instantaneous nor linear; rather, they depend on the successful diffusion of robotic technologies cross industries and the resulting improvements in production efficiency. These findings are consistent with the theoretical framework in Sect. 2.4 and undesrscore that the carbon-mitigating role of robotics manufacturing is cumulative and conditional on sustained industrial development.

**Table 5:** Results of the mechanism tests.

	(1)	(2)	(3)	(4)	(5)	(6)	(7)	(8)	(9)
	Panel A: Whole period			Panel B: Left of the turning point (Early stage)			Panel C: Right of the turning point (Mature stage)		
Explained variable	lnad	EE	lnCO2	lnad	EE	lnCO2	lnad	EE	lnCO2
lnSRobot	0.457^***^	−2.876^**^	0.054^***^	0.020	−0.214	−0.016	0.448^**^	3.730	−0.048
	(0.052)	(1.219)	(0.015)	(0.141)	(1.972)	(0.031)	(0.227)	(2.492)	(0.040)
(lnSRobot)^2^		1.208^***^	−0.029^***^						
		(0.335)	(0.005)						
lnadoption		1.310^***^	−0.039^***^		1.846^***^	−0.055^***^		1.330^***^	−0.000
		(0.503)	(0.010)		(0.408)	(0.012)		(0.429)	(0.007)
EE			−0.007^*^			−0.005^*^			−0.022^***^
			(0.004)			(0.003)			(0.002)
Control variables	YES	YES	YES	YES	YES	YES	YES	YES	YES
City fixed effect	YES	YES	YES	YES	YES	YES	YES	YES	YES
Year fixed effect	YES	YES	YES	YES	YES	YES	YES	YES	YES
Extreme point		1.190	0.916						
t-value of the U-test		12.36***	3.55***						
N	3,324	3,324	3,324	1,915	1,915	1,915	1,409	1,409	1,409
R^2^	0.858	0.509	0.932	0.814	0.530	0.910	0.948	0.719	0.968
Bootstrap test (indirect effect)	−0.0029			−0.0033			−0.0096		
BC 95% CI	[−0.28134, −0.00028]			[−0.01552, −0.00008]			[−0.44656, −0.00523]		

In panel B, the bootstrap-based indirect effect does not provide a valid evaluation of the transmission channel, as the prerequisite relationship between lnSRobot and lnadoption is not significant. Robust standard errors in parentheses, * *p* < 0.1, ** *p* < 0.05, and *** *p* < 0.01.

### Heterogeneity across regions and subsectors

#### Stronger and earlier carbon-reduction effects in the central and western regions

Considering the pronounced regional disparities in economic development and industrial structure in China, the spatial distribution and environmental effects of robotics manufacturing are likely to be heterogeneous across regions. Robotics manufacturing activities and their associated supply chains are more densely concentrated in the eastern region, whereas the central and western regions are still in earlier stages of industrial clustering and technological upgrading^34^. These differences imply that the marginal environmental impact of robotics manufacturing may vary systematically across regions.

To examine this heterogeneity, we re-estimate the baseline model separately for the eastern, central, and western regions. The results reported in Table 6 reveal clear regional differences. In the eastern region, the quadratic term of $\:{lnSRobot}_{it}$ is statistically insignificant, indicating the absence of a nonlinear relationship between robotics manufacturing and carbon emissions. Despite the region’s relatively advanced robotics industry, further expansion of manufacturing scale does not generate a distinct carbon-reduction turning point. This suggests that in highly industrialised regions, the environmental benefits of robotics manufacturing are increasingly constrained by existing production scale and energy demand, and further emission reductions rely more on qualitative technological improvements than on scale expansion.

In contrast, the central region exhibits a statistically significant inverted U-shaped relationship between robotics manufacturing and carbon emissions. The estimated turning point (0.943) is substantially lower than that of the national sample (1.342), indicating that the carbon-reduction effects of robotics manufacturing emerge at an earlier stage. In the western region, although the quadratic term is statistically significant and suggests pronounced nonlinear effects, the U-test (p-value = 0.161) does not formally confirm a well-defined inverted U-shaped relationship. Nevertheless, the relatively low estimated turning point (0.328) implies that incremental expansion of robotics manufacturing from a low development base may still generate considerable marginal environmental benefits.

Taken together, these patterns are broadly consistent with the technological leapfrogging hypothesis, whereby late-developing regions are able to adopt more advanced and energy-efficient production technologies directly, bypassing the high-carbon trial-and-error phase experienced by early industrialisers. As robotics manufacturing clusters gradually form in the central and western regions, technological spillovers and supply-chain specialisation begin to improve production efficiency, while lower baseline emission levels and a higher availability of clean energy sources—such as wind, solar, and hydropower—further amplify the potential environmental benefits. As a result, the expansion of robotics manufacturing in the central, and potentially the western, regions can yield stronger marginal efficiency gains and accelerate the transition toward carbon reduction.

Overall, the regional heterogeneity results suggest that while the eastern region faces diminishing marginal environmental returns from further robotics manufacturing expansion, the central and western regions possess greater potential to achieve earlier and more pronounced carbon-reduction effects through technological upgrading. These findings complement the baseline and mechanism analyses by highlighting the importance of regional development stages in shaping the environmental outcomes of robotics manufacturing.

**Table 6:** Regional heterogeneity tests.

	(0)	(1)	(2)	(3)
	Whole	Eastern	Central	Western
lnSrobot	0.054^***^	0.065^***^	0.102^***^	0.031
	(0.014)	(0.022)	(0.025)	(0.031)
(lnSRobot)^2^	−0.020^***^	−0.000	−0.054^***^	−0.047^***^
	(0.004)	(0.006)	(0.008)	(0.007)
Control variables	YES	YES	YES	YES
City fixed effect	YES	YES	YES	YES
Year fixed effect	YES	YES	YES	YES
N	3,324	1,020	924	984
R^2^	0.926	0.939	0.918	0.909
Extreme point	1.342	NA	0.943	0.328
t-value of the U-test	3.89***	NA	4.16***	0.99

Robust standard errors in parentheses, * *p* < 0.1, ** *p* < 0.05, and *** *p* < 0.01.

#### System integration delivers stronger carbon-reduction effects than ontology manufacturing

Enterprises located at different positions along the robotics manufacturing value chain engage in heterogeneous production activities, leading to systematic differences in technological content, energy use, and environmental impact. Within the robotics manufacturing industry, ontology manufacturing and system integration represent two key downstream segments with distinct characteristics. Ontology manufacturing primarily involves hardware design, machining, and component assembly, which are relatively material- and energy-intensive and thus associated with higher direct emissions. By contrast, system integration focuses on software development, control systems, and application-oriented solutions for downstream users, relying less on heavy industrial processing and exhibiting greater potential for efficiency gains.

To capture these differences empirically, we separately examine ontology manufacturing and system integration enterprises at the city level. The results reported in Table 7 show that both subsectors exhibit a statistically significant inverted U-shaped relationship between robotics manufacturing and carbon emissions. However, notable differences arise in the timing of the emission-reduction effects. The estimated turning point for system integration (1.211) is lower than that for ontology manufacturing (1.239) and the overall industry (1.342), indicating that the carbon-reduction effects of system integration materialise at an earlier stage of development.

These findings are consistent with the “smile curve” perspective, which suggests that higher value-added activities located at the upstream and downstream ends of the value chain—such as system integration—tend to generate greater efficiency gains than midstream manufacturing activities. While ontology manufacturing remains closely tied to energy-intensive physical production, system integration enhances process coordination, resource allocation, and intelligent optimisation, thereby facilitating energy savings both within the robotics industry and in downstream user sectors.

Overall, the subsector heterogeneity analysis indicates that although both segments eventually contribute to carbon mitigation as the robotics industry matures, system integration delivers earlier and more pronounced carbon-reduction effects than ontology manufacturing. These results highlight the importance of value-chain positioning in shaping the environmental impact of robotics manufacturing and complement the baseline and mechanism analyses.

**Table 7:** Subsector heterogeneity tests.

	(0)	(1)	(2)
	Whole	Ontology manufacturing	System integration
lnSrobot	0.054^***^	0.063^***^	0.049^***^
	(0.014)	(0.016)	(0.014)
(lnSrobot)^2^	−0.020^***^	−0.025^***^	−0.020^***^
	(0.004)	(0.005)	(0.003)
Control variables	YES	YES	YES
City fixed effect	YES	YES	YES
Year fixed effect	YES	YES	YES
N	3,324	3,324	3,324
R^2^	0.926	0.926	0.926
Extreme point	1.342	1.239	1.211
t-value of the U-test	3.89***	4.01***	3.48***

Robust standard errors in parentheses, * *p* < 0.1, ** *p* < 0.05, and *** *p* < 0.01.

## Conclusions and policy implications

This study confirms the conjecture proposed in the Introduction that the environmental impact of the robotics manufacturing industry is nonlinear rather than monotonic. By shifting the analytical focus from robot applications to the manufacturing stage—a dimension largely neglected in the existing literature—this study fills an important research gap and provides a more complete assessment of the environmental consequences of robotics development. Using panel data on 28,624 robotics manufacturers across 277 Chinese cities, we identify a robust inverted U-shaped relationship between robotics manufacturing development and urban carbon emissions. Carbon emissions rise in the early stage of industry expansion but decline after the industry reaches a moderate scale, consistent with a dynamic transition from a scale-dominated phase to a technology- and diffusion-driven phase.

Mechanism tests further support the sequential pathway proposed in Sect. 2.4: robotics manufacturing promotes robot adoption, which improves urban energy efficiency, and ultimately reduces carbon emissions. Importantly, this transmission mechanism is stage-dependent—it becomes statistically operative only after the industry passes the turning point, whereas it is not validated in the early stage when the prerequisite link from manufacturing expansion to adoption is insignificant. Heterogeneity analysis shows that carbon-reduction effects are more pronounced in the central region, whereas no clear mitigation effect is identified in the eastern region, suggesting a potential latecomer advantage in green industrialization. Subsector results show that system integration achieves an earlier turning point and stronger carbon-reduction potential than ontology manufacturing, consistent with the idea that higher value-added segments deliver greater efficiency spillovers.

The identification of an inverted U-shaped relationship implies that robotics manufacturing exhibits a stage-dependent environmental effect, and thus requires dynamic and targeted policy interventions rather than uniform support measures.

First, based on the estimated inflection point, environmental protection authorities and industry and information technology departments should jointly establish a robotics industry cluster carbon-emission monitoring platform. This platform should track cities whose number of robotics manufacturing enterprises is approaching the critical threshold and provide early warning signals. Targeted low-carbon technology assistance—such as energy-efficiency diagnostics, cleaner production consulting, and access to green financing—can help these cities cross the inflection point more rapidly and avoid prolonged high-emission lock-in.

Second, policy design should reflect regional heterogeneity. In the eastern region, where the robotics industry is more mature and carbon pressures remain relatively high, the policy focus should shift from investment attraction toward green innovation competition, for example through robot low-carbon technology patent awards or performance-based subsidies tied to emission reductions. In contrast, the central and western regions should adopt a green industry transfer and acceptance guideline, explicitly linking robotics park construction with clean energy supply, environmental access standards, and technology-transfer requirements, so as to leverage their latecomer advantage for low-carbon industrial upgrading.

Third, industrial policies should prioritise system integration, given its earlier and stronger carbon-reduction effects. Governments can introduce a carbon-reduction benefit certification scheme for system integrators and use certification outcomes as a criterion for priority procurement, green credit, and demonstration projects, thereby amplifying downstream efficiency spillovers.

Several limitations should be acknowledged. First, although fixed effects and instrumental variable approaches are employed, we cannot fully rule out endogeneity arising from unobservable, time-varying city-level factors, such as changes in local leadership preferences or environmental governance intensity. Second, our evidence is based on Chinese cities, and the generalizability of the findings to other institutional contexts requires further validation. Future work could incorporate richer firm-level indicators (e.g., output, patents, energy use), explicitly model policy and regulatory intensity, and extend the analysis to other countries or multi-country settings, while also considering broader life-cycle or supply-chain emissions where data permit.

## Supplementary Information

Below is the link to the electronic supplementary material.

Supplementary Material 1

## Data Availability

The datasets used and analysed during the current study are available from the corresponding author on reasonable request.

## Footnotes

1 https://www.iea.org/reports/co2-emissions-in-2022.

2
https://www.gov.cn/zhengce/zhengceku/2021-12/03/content_5655701.htm.

3 This study uses the *Top 100 Chinese Robotics Manufacturing Enterprises* as a benchmark to identify their industry code distribution from the CNECIPS database. Based on the *Industrial Classification for National Economic Activities* (GB/T 4754–2017), we define 14 core industry categories closely related to the robotics industry, covering manufacturing and information technology services. Firm-level data are then extracted by restricting the sample to these categories and applying *“robotics manufacturing”* as a core keyword to ensure accurate identification.
