# Supplementary material for: Exploring the nonlinear relationship between robotics manufacturing and urban carbon emissions
Source: Sci Rep. 2026 Apr 1;16:15646. doi: 10.1038/s41598-026-46922-y (PMC13187413; doi:10.1038/s41598-026-46922-y)
Supplement: Supplementary file 1 — Supplementary Material 1 [file 41598_2026_46922_MOESM1_ESM.docx]

**Exploring the Nonlinear Relationship between Robotics Manufacturing and Urban Carbon Emissions**

Funding declaration

*This work was supported by the Guangdong Provincial Committee for Basic and Applied Basic Research Fund (2024A1515110058).*
